# Blinking Bug Bite

**DOI:** 10.5811/cpcem.2018.9.39466

**Published:** 2018-10-09

**Authors:** Joseph Miller, Mark Walsh, Jessica Magyar, Nuha Zackariya, John Rice, Katherine Rice, Dawn Ziegelmaier, Patrick Hanlon, Joseph Dynako, David Zimmer, Faadil Shariff, Michael T. McCurdy, Shane Kappler, Paul Guentert

**Affiliations:** * Henry Ford Hospital, Department of Emergency Medicine, Detroit, Michigan; † St. Joseph Regional Medical Center, Department of Emergency Medicine, Mishawaka, Indiana; ‡ Indiana University, Bloomington, Indiana; § South Bend Clinic, Michiana Pediatrics, South Bend, Indiana; ¶ Indiana University School of Medicine, South Bend, Indiana; || University of Maryland School of Medicine, Department of Emergency Medicine, Baltimore, Maryland; # Cambridge Health Alliance, Cambridge, Massachusetts; ** Pulmonary and Critical Care Associates PC, South Bend, Indiana

## CASE PRESENTATION

**Image 1:**
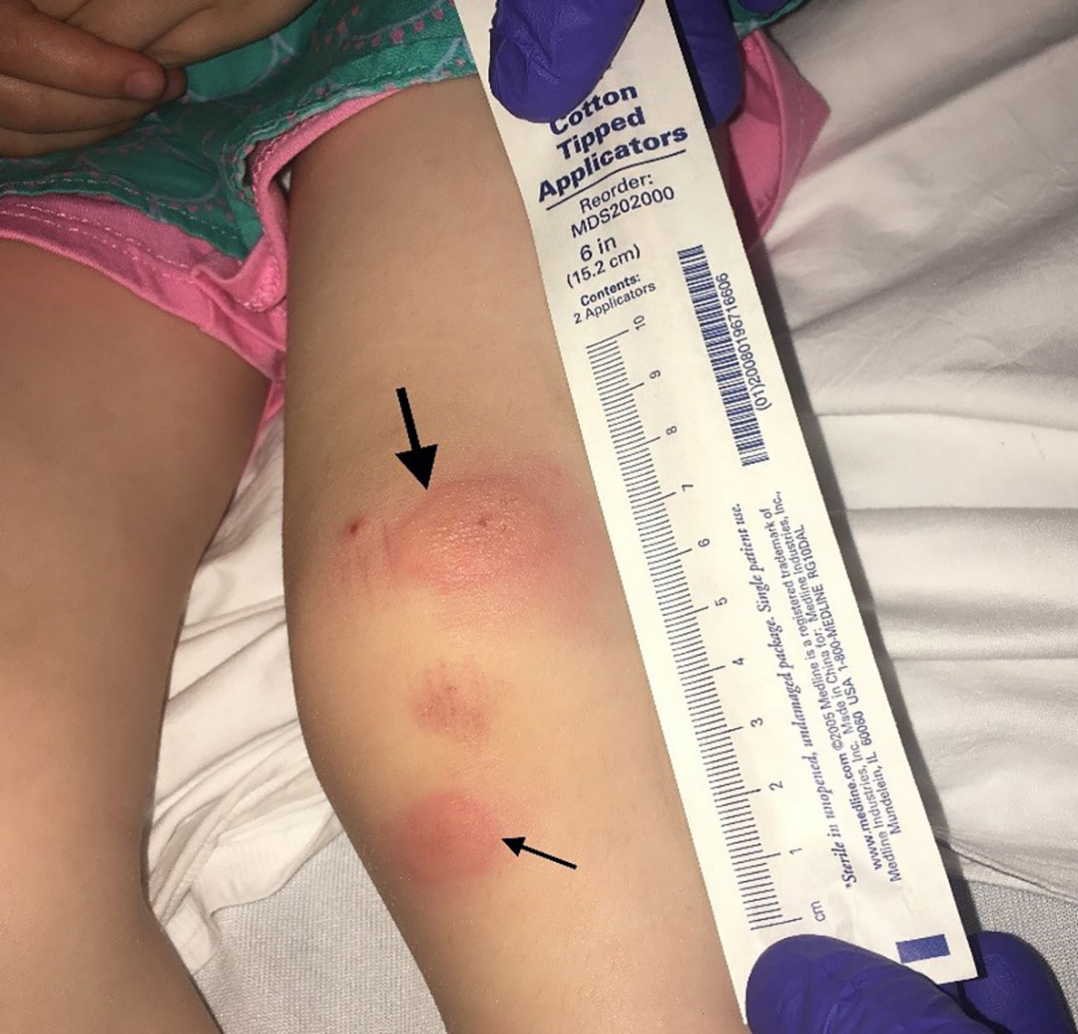
The insect bite skin lesions on the knee varying in size (arrows).

**Image 2:**
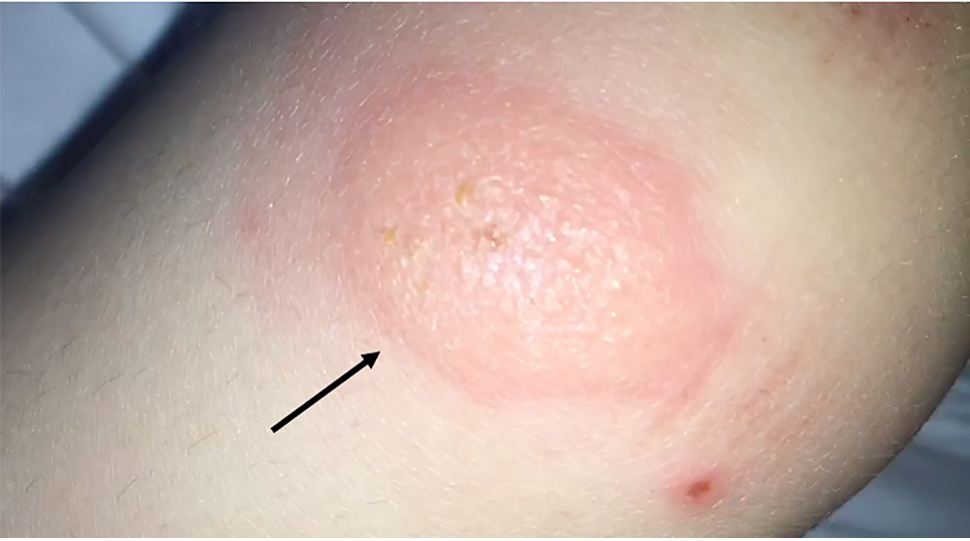
Close-up of skin lesion (arrow).

A three-year-old female presented to a community emergency department with a one-day pruritic rash on her knee. The patient and the parents noted that the rash blanched intermittently and that this blanching appeared to be what they called a “blinking” bug bite. Physical examination revealed a normal child with no heart murmur and two bullous lesions around the left knee that blanched in a pulsatile fashion, corresponding to the femoral pulse (Images 1 and 2, Video).

## DIAGNOSIS

A review of the medical literature revealed a single “Images in Clinical Medicine” from the *New England Journal of Medicine*, which revealed a severe dermatitis of the lower extremities that had similar presentation.^1^ No other cases were noted in the literature. The wounds were anesthetized and debrided for concern of a staph infection. The patient was placed on antibiotics and healed uneventfully. The case presented here represents an example of a physical manifestation of Quincke’s sign, not related to aortic insufficiency but to the rarely-noted effect of intense arterial dilatation in the bullous inflammation of the affected subcutaneous area. Quincke’s pulse is a physical finding of aortic insufficiency and, as in this case, focal arterial dilatation. Here, the arterial dilatation in the area of the bite led to an inability of arterioles to maintain sufficient pressure during diastole, resulting in the pulsating blanching and flushing that produced a “blinking” bug bite.

**CPC-EM Capsule**

What do we already know about this clinical entity?

Google Scholar and PubMed reveal only one case report of a “blinking” bug bite. However, searchable online blog posts reveal similar descriptions of these bites.

What is the major impact of the image(s)?

The images here demonstrate a classic example of the “blinking” bug bite, which has not been well described in the medical literature.

How might this improve emergency medicine practice?

These images and video may act as a reference for emergency physicians to more easily diagnose this benign condition.

Documented patient informed consent and/or Institutional Review Board approval has been obtained and filed for publication of this case report.

## Supplementary Information

Video This recording demonstrates the blinking bug bite, a manifestation of Quincke’s pulse.
